# Pulsatile vessel causing Esophageal compression and dysphagia: a rare complication of anterior cervical discectomy and fusion

**DOI:** 10.1093/omcr/omag182

**Published:** 2026-10-04

**Authors:** Obada Daaboul, Muhammed Yaman Swied, Muhammad Anas Abdulrazzak, Muaataz Azzawi, Ramprasad Jegadeesan

**Affiliations:** Department of Internal Medicine, Southern Illinois University School of Medicine, 701 North First Street, Springfield, Sangamon County, Illinois 62702, United States of America; Department of Internal Medicine, Southern Illinois University School of Medicine, 701 North First Street, Springfield, Sangamon County, Illinois 62702, United States of America; Department of Internal Medicine, Southern Illinois University School of Medicine, 701 North First Street, Springfield, Sangamon County, Illinois 62702, United States of America; Department of Internal Medicine, Southern Illinois University School of Medicine, 701 North First Street, Springfield, Sangamon County, Illinois 62702, United States of America; Digestive Care Physicians, 11315 Johns Creek Parkway, Suite 400, Johns Creek, Georgia 30097, United States of America

**Keywords:** dysphagia, ACDF, extrinsic esophageal compression, pulsatile vessel

A 75-year-old man with cervical radiculopathy underwent C3—T1 anterior cervical discectomy and fusion (ACDF). Three weeks later, he developed dysphagia with a globus sensation. Computed tomography angiography (CTA) of the chest was obtained given mild tachycardia and concerns for pulmonary embolism which revealed distal esophageal wall thickening without other vascular anomalies.

Esophagogastroduodenoscopy (EGD) revealed esophageal narrowing at 20 cm from the incisors caused by extrinsic compression from a pulsating lesion (Fig. 1). In this context, postoperative soft-tissue edema with adjacent vascular pulsatility was considered. However, comparison of pre- and post ACDF chest CT with radiology revealed no significant anatomic changes. The displaced vessel origin or precise anatomical basis of the pulsatile compression was not clearly identifiable on CT; after discussion with radiology, the consensus was that this likely represented a subtle dynamic change that was difficult to appreciate on cross-sectional imaging but was apparent endoscopically, and therefore, the EGD findings and patient symptoms were most consistent with post-ACDF soft tissue edema accentuating adjacent normal vascular pulsatility. Differential considerations included esophageal retraction, prominence of the cervical plate, and prevertebral swelling; in our case, the focal pulsatile extrinsic narrowing on EGD supported extrinsic mass effect from adjacent vascular pulsatility. The patient was managed conservatively with swallow precautions and observation, and his symptoms resolved spontaneously within several weeks, as he reported on a follow-up visit several weeks later.

**Figure 1:**
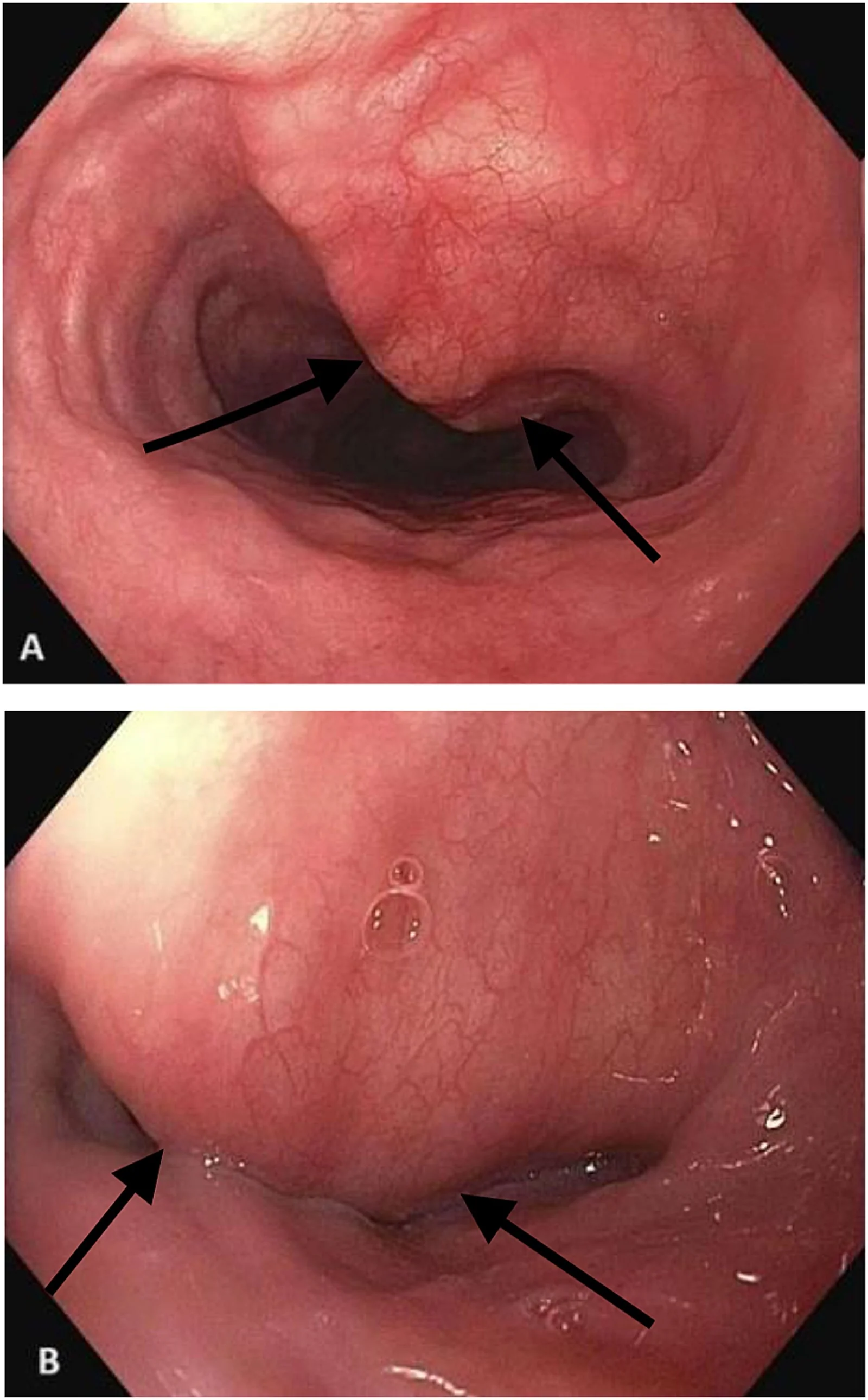
Endoscopic images showing a pulsatile vessel compressing the esophagus at 20 cm from the incisors. A: During diastole B: During systole.

ACDF is the most common procedure for degenerative cervical spine disease [1]. Postoperative Dysphagia is a well-recognized complication of ACDF reported in approximately 71% of patients and may persist in 12-14% after one year of the procedure [1, 2]. proposed etiologies are multifactorial and include the mechanisms noted above; however, dysphagia due to extrinsic esophageal narrowing by a pulsatile vessel is rarely reported [3]. Because ACDF-specific diagnostic guidelines for postoperative dysphagia are not standardized, we suggest a stepwise evaluation: (1) screen for red-flag features (airway compromise, rapidly progressive neck swelling, inability to tolerate secretions, fever/sepsis) and obtain urgent spine/ENT assessment with cross-sectional imaging to exclude postoperative complications; (2) in clinically stable patients with persistent symptoms, proceed with EGD to evaluate for esophageal pathology and/or extrinsic impingement. This case emphasizes a benign, self-limiting cause of postoperative dysphagia after ACDF to raise awareness and reduce unnecessary diagnostics and resource use.

## Supplementary Material

Video_omag182
